# Oral Delivery of mRNA Vaccine by Plant-Derived Extracellular Vesicle Carriers

**DOI:** 10.3390/cells12141826

**Published:** 2023-07-11

**Authors:** Margherita A. C. Pomatto, Chiara Gai, Federica Negro, Lucia Massari, Maria Chiara Deregibus, Francesco Giuseppe De Rosa, Giovanni Camussi

**Affiliations:** 1 EvoBiotech s.r.l., 10122 Turin, Italy; cgai@evobiotech.it (C.G.);; 2 Department of Medical Science, University of Turin, A.O.U. Città della Salute e della Scienza di Torino, 10126 Turin, Italy

**Keywords:** oral, capsule, extracellular vesicles, plant, delivery, carrier, mRNA, vaccine, SARS-CoV-2

## Abstract

mRNA-based vaccines were effective in contrasting SARS-CoV-2 infection. However, they presented several limitations of storage and supply chain, and their parenteral administration elicited a limited mucosal IgA immune response. Extracellular vesicles (EVs) have been recognized as a mechanism of cell-to-cell communication well-preserved in all life kingdoms, including plants. Their membrane confers protection from enzyme degradation to encapsulated nucleic acids favoring their transfer between cells. In the present study, EVs derived from the juice of an edible plant (*Citrus sinensis*) (oEVs) were investigated as carriers of an orally administered mRNA vaccine coding for the S1 protein subunit of SARS-CoV-2 with gastro-resistant oral capsule formulation. The mRNA loaded into oEVs was protected and was stable at room temperature for one year after lyophilization and encapsulation. Rats immunized via gavage administration developed a humoral immune response with the production of specific IgM, IgG, and IgA, which represent the first mucosal barrier in the adaptive immune response. The vaccination also triggered the generation of blocking antibodies and specific lymphocyte activation. In conclusion, the formulation of lyophilized mRNA-containing oEVs represents an efficient delivery strategy for oral vaccines due to their stability at room temperature, optimal mucosal absorption, and the ability to trigger an immune response.

## 1. Introduction

Great advancements in the development of mRNA-based vaccines were achieved after the SARS-CoV-2 pandemic. The technology of mRNA transfer was first suggested by Wolff et al. [1], and remained for many years under development. The SARS-CoV-2 infection accelerated mRNA vaccine research allowing surprising speed in development, regulatory approval, and massive production of efficient vaccines. This approach was found to be effective and safe, allowing faster adaptability than conventional vaccines by inducing the production of viral antigens directly within the recipient and therefore triggering an immune response [2]. Progress in nanotechnology allowed the improvement of non-viral vectors for the delivery of mRNA vaccines. These vectors based on lipid and polymeric nanoparticles encapsulating mRNA may provide protection from enzyme degradation and an efficient delivery without, however, the risk of preexisting immunity against the vector itself [3,4]. Critical advancements in the mRNA-based vaccines were achieved by optimizing mRNA sequences and the strategies of delivery [5,6]. However, several logistic difficulties related to storage, stability, and distribution still remain and have limited the diffusion of mRNA vaccines in underdeveloped countries. Moreover, most SARS-CoV-2 vaccines in development are intramuscularly administered with the necessity of needles and healthcare workers [7]. Nonetheless, the parenteral administration triggered an inefficient mucosal immunoglobulin A (IgA)-based immune response [8,9]. This stimulated the search of new strategies for the development of mucosal vaccines against SARS-CoV-2 [7,10]. The oral administration can solve these difficulties by eliciting an immune response at the intestine level, where the abundant production of IgA by plasma cells confers local and systemic mucosal protection [11]. It has been suggested, for instance, that edible algae could represent a promising approach for the production and oral delivery of a SARS-CoV-2 RBD immunogen [12]. Recently, a bacteria-based strategy was successfully adopted to orally deliver mRNA vaccine for SARS-CoV-2 using *Salmonella* as carrier of a virus replicon-based mRNA vaccine [13].

Extracellular vesicles (EVs) are emerging as promising delivery systems for nucleic acid therapies. EVs have been originally described in eukaryotes as a mechanism of cell-to-cell communication. It has been subsequently recognized that this mechanism of communication is well-preserved in all life kingdoms, including prokaryotes and plants [14]. Interestingly, EVs allow horizontal transfer of vesicle-encapsulated nucleic acids, and their lipid bilayer membrane confers protection from degradation. It was found that engineered EVs containing an exogenous mRNA coding for the green fluorescent protein were able to induce protein translation in target cells [15]. Recently, bacteria-derived EVs coated with spike receptor-binding domain were shown to elicit an immune response following intranasal administration [16]. Moreover, EVs released from engineered mammalian cells to express SARS-CoV-2 S antigen were shown to efficiently stimulate CD8+ T immune response [17].

Among EVs, plant-derived EVs are a non-toxic extractive product from a natural, abundant, and edible source. With respect to mammalian EVs, they have the advantage of scalable production and the delivery of a wide range of drugs [18]. We recently demonstrated the efficacy of EVs isolated from orange (*Citrus sinensis*) juice (oEVs) as carriers of an mRNA-based vaccine in soluble formulation using multiple administration routes (intramuscular, oral, and intranasal) in mice [19]. In the present study, we investigated the use of EVs derived from edible plants as a carrier for the oral delivery of a SARS-CoV-2 mRNA vaccine formulated as an easy-to-use oral capsule. For that, oEVs containing mRNA coding for S1 SARS-CoV-2 antigen were lyophilized and encapsulated in gastro-resistant capsules and orally administered to rats. The resistance of EVs and mRNA was evaluated over time until one year, and the ability of the treatment to induce animal immunization was investigated.

## 2. Materials and Methods

### 2.1. Treatment Production

The oEVs were isolated from freshly squeezed orange juice from *C. sinensis*, type Tarocco, organic (Arancebio srl, Francofonte, SR, Italy) collected in January 2021 and 2022, following the procedure previously described [19]. Briefly, the juice was manually filtered with a strainer and centrifuged at 4000× *g* for 30 min. The supernatant was collected and consequently ultracentrifuged at 10,000× *g* for 1 h at +4 °C, and then at 100,000× *g* for 2 h at +4 °C (Optima L-90K ultracentrifuge, rotor 45 Ti, polycarbonate tubes, Beckman Coulter, Milan, Italy). The pellet was re-suspended in saline solution (NaCl 0.9%, B. Braun, Melsungen, Germany) added with 1% DMSO (Sigma-Aldrich, Merck, Darmstadt, Germany), sterilized by filtration with 0.22 µm syringe filters (Millex, Millipore, Merck, Darmstadt, Germany), and stored at −80 °C for further experiments.

The oEVs were loaded with mRNA using a proprietary technique reported in the patent application WO/2022/152771A1, as previously described [19]. Briefly, 6 × 10^12^ oEV/mL were mixed with mRNA and 0.2% *w/v* cation proteins and subjected to osmotic stress to allow mRNA encapsulation. Then, loaded oEVs were washed via centrifugation at 100,000× *g* for 2 h at +4 °C (Optima L-90K ultracentrifuge, rotor 45 Ti, polycarbonate tubes, Beckman Coulter, Milan, Italy), and no adjuvants were added to the formulation to improve immunization effect. The mRNA sequence of the surface glycoprotein, Spike-S1-RBD protein was designed as previously described [19], based on the genomic sequence of SARS-CoV-2, NC_045512.2, covering ORF2 (nucleotide position 22518–23186, 669 nt). The mRNA was purchased from RiboPro (Oss, Netherlands) with a 5′UTR designed for high expression, a poly-A tail, and a Cap1 with methylation, but without codon optimization or other mRNA modifications. To avoid innate immune-mediated translational repression, dsRNA was removed. After loading, oEV were freeze-dried with a benchtop freeze dryer (FreeZone™ 2.5 L −50 °C, Labconco, Kansas City, MO, USA) and used to fill gelatin capsules size 9 (Torpac Europe, Heerlen, The Netherlands) using the kit provided by the manufacturer. Then, capsules were coated with Eudragit 4% and 21% coating solutions, prepared in accordance with the manufacturer’s instructions (Eudragit L100, Evonik, Darmstadt, Germany), and stored at room temperature (RT) for experiments. For mRNA and EV resistance analysis, over time, capsules were opened, and their content was reconstituted with water to reach the starting volume before analysis at each time point. Each analysis of treatments was conducted on three independent experiments.

### 2.2. Particle Characterization with NTA Analysis

The oEVs’ sizes and concentrations were analyzed by nanoparticle tracking analysis (NTA) with the ZetaView TWIN Laser System, equipped with Zeta View software version 8.05.14 SP7 (ParticleMetrix, Inning am Ammersee, Germany). For each sample, oEVs were diluted in a range of 1:2000–1:10,000 in 5–10 mL of saline solution (NaCl 0.9%, B. Braun, Melsungen, Germany) previously filtered with 0.1 μm membranes (Millex, Millipore, Merck, Darmstadt, Germany). The analysis was repeated in three independent experiments.

### 2.3. Particle Analysis with TEM Technique

The analysis of the oEV integrity and morphology was performed using transmission electron microscopy (TEM), as previously described [19]. Briefly, oEVs were left to adhere for 20 min on 200 mesh nickel formvar carbon-coated grids (Electron Microscopy Science, Hatfield, PA, USA). Subsequently, grids were incubated with 2.5% glutaraldehyde plus 2% sucrose and washed with distilled water. Finally, samples were negatively stained with Nano-Van and Nano-W (Nanoprobes, Yaphank, NY, USA) and acquired with Jeol JEM 1400 Flash electron microscope (Jeol, Tokyo, Japan). The analysis was repeated in three independent experiments.

### 2.4. S1 mRNA Analysis with qRT-PCR Assay

To directly compare the S1 mRNA amounts in samples, total RNA was isolated from the same volume of sample with miRNeasy mini kit (Qiagen, Hilden, Germany), following the manufacturer’s instructions as previously described [19]. For each sample, RNA was eluted in 50 µL of nuclease-free water (Ambion, Thermo Fisher Scientific, Waltham, MA, USA). The RNA concentration was measured as the absorbance at 260 nm with a spectrophotometer (mySPEC, VWR, Radnor, PA, USA), and samples were stored at −80 °C until analysis.

The retro-transcription from RNA to cDNA was performed on 10 µL of RNA using a High-Capacity cDNA Reverse Transcription Kit (Thermo Fisher Scientific, Waltham, MA, USA) following the manufacturer’s instructions. Syn-cel-miR-39 (Qiagen, Hilden, Germany) was added as spike-in for normalization, and samples were subjected to 1.8 dilution with nuclease-free water (Ambion, Thermo Fisher Scientific, Waltham, MA, USA).

The qRT-PCR was run in triplicate on each sample. Primers (Eurofins Genomics, Milan, Italy) are listed in Table S1, and the universal primer (Qiagen, Hilden, Germany) was used as the reverse primer for syn-cel-miR-39. In each reaction, 2.5 µL of diluted cDNA were combined with SYBR GREEN PCR Master Mix (Thermo Fisher Scientific, Waltham, MA, USA), as described by the manufacturer’s protocol. The Real-Time Thermal Cycler Quant Studio 12k and ExpressionSuite Software 1.0.3 (Thermo Fisher Scientific, Waltham, MA, USA) were used to calculate relative quantification (RQ) values via the 2^−ΔΔCt^ method. The analysis was repeated in three independent experiments.

### 2.5. S1 mRNA Investigation Using PCR Technique

As previously described [19], cDNA was amplified with iProofTM High-Fidelity DNA Polymerase (Biorad, Hercules, CA, USA) following the manufacturer’s instructions. The components of the PCR reaction mix were 4 ng cDNA, 500 nM Primer (see Table S1, Eurofins Genomics, Milan, Italy), 0.5 μL iProof DNA Polymerase (Biorad, Hercules, CA, USA), 10 μL 5x iProof HF buffer, 1 μL dNTPs, 0.5 μL MgCl_2_, and nuclease-free water (Thermo Fisher Scientific, Waltham, MA, USA). The final reaction volume was 50 μL. The CTR DNA template and 1.3 kB primers provided by the kit were amplified as an internal amplification control. The PCR was run with VERITI Thermal Cycler (Thermo Fisher Scientific, Waltham, MA, USA), and 30 amplification cycles were set.

For gel electrophoresis, 3 μL 6X TriTrack DNA Loading Dye from GeneRuler 100 bp Plus DNA Ladder kit (Thermo Fisher Scientific, Waltham, MA, USA) were mixed with 15 μL PCR products, and 15 μL of the mix were loaded on a 5% Mini-PROTEAN^®^ TBE Gel (Biorad, Hercules, CA, USA). The run was performed with 1X TBE buffer (Biorad, Hercules, CA, USA) at 100 V for 45 min. Gels were stained with a 0.5 μg/mL ethidium bromide (Biorad, Hercules, CA, USA) solution and subsequently washed in sterile water (B. Braun, Milan, Italy). Images were acquired with ChemiDoc System (Biorad, Hercules, CA, USA). Data were obtained from three independent experiments. The analysis was repeated in three independent experiments.

### 2.6. Rat Immunization and Sample Collection

This study was conducted at Takis s.r.l. (Castel Romano, Rome, Italy), and performed in accordance with the European Directive 2010/63/EU on the protection of animals used for scientific purposes, applied in Italy by the Legislative Decree 4 March 2014, n.26. This study was included in a main research project approved by the Italian Ministry of Health.

Eleven female Sprague Dawley rats, internally bred by Takis, were used for the study. Before immunization, on day 0, rats were subjected to blood sampling (to be processed as serum) under anesthesia by inhalation of isoflurane, following registration of body weight. Immunization was performed by administering capsules containing empty oEVs (n = 5) and mRNA-loaded oEVs (oEV-S1, n = 6) orally, using a gavage needle. Each capsule contained 1.2 × 10^12^ oEVs containing 100 µg mRNA. Rats received one capsule per day on days 1, 2, and 3 and boost immunizations after 3 weeks on days 23, 24, and 25. During the study, rats were monitored daily for clinical signs, mortality, or morbidity. Body weight was recorded on days 0, 7, 14, 21, 28, 35, and 42. Rats were euthanized after 2 weeks from the last boost immunization, at day 42. Rats were subjected to anesthesia and blood sampling (to be processed as serum) and euthanized. Sera were stored at −20 °C until use. Representative and clean gut portions of the jejunum, duodenum, ileum, and colon were fixed in formalin solution and preserved at 4 °C until use. Spleens were collected in culture media and kept at 4 °C until cell isolation. For that, spleens were immediately disrupted and filtered through a 40 μm cell strainer (PluriSelect, Leipzig, Germany), diluted with PBS (Euroclone, Milan, Italy), and centrifuged at 500× *g* at 4 °C for 5 min. Each pellet was resuspended in 2 mL cold RBC lysis buffer 1X (Thermo Fisher Scientific, Waltham, MA, USA) and incubated on ice for 5 min to lyse red blood cells. The reaction was stopped with 10 mL ice-cold PBS, and cells were pelleted at 400× *g* at 4 °C for 5 min and resuspended in RPMI 1640 supplemented with 10% FBS (Euroclone, Milan, Italy). Data presented refer to n = 5 for rats treated with oEVs and n = 6 for rats treated with mRNA-loaded oEVs.

### 2.7. Antibody Titer Measurement

Enzyme-linked Immunosorbent Assay (ELISA) was used to detect the presence of specific IgG, IgM, and IgA antibodies against the S1 protein of the SARS-CoV-2 virus in sera of immunized rats. For that, 100 μL/well of 1 μg/mL SARS-CoV-2 S1 RBD recombinant protein (RP-87706, Thermo Fisher Scientific, Waltham, MA, USA) was used to coat Nunc Maxisorp ELISA plates (Thermo Fisher Scientific, Waltham, MA, USA) overnight at 4 °C after dilution in BupH Carbonate–Bicarbonate Buffer (Thermo Scientific, Waltham, MA, USA). Plates were washed three times with PBS 1X, blocked with PBS 1X-3% bovine serum albumin (BSA, Sigma-Aldrich, Merck, Darmstadt, Germany) for 1 h at 37 °C, washed five times with PBS 1X-0.05% Tween20 (Sigma-Aldrich, Merck, Darmstadt, Germany), and incubated with 100 µL/well of different serial dilutions of samples in duplicate (starting from dilution 1:50) for 1 h at 37 °C. Dilution buffers (PBS 1X-0.05% Tween20-3% BSA for IgG and IgA, PBS 1X-3% BSA-5% FBS for IgM) were also used as blank. Plates were washed with PBS 1X-0.05% Tween20 five times and incubated for 1 h at RT with a secondary antibody for IgG (1:5000, 31,471, Invitrogen, Thermo Fisher Scientific, Waltham, MA, USA), IgM (1:5000, 31476, Invitrogen, Thermo Fisher Scientific, Waltham, MA, USA); or IgA (1:1000, 84,707, Invitrogen, Thermo Fisher Scientific, Waltham, MA, USA). After several washes with PBS 1X-0.05% Tween20, plates were incubated with TBM Stabilized Chromogen (Life Technologies, Thermo Fisher Scientific, Waltham, MA, USA) for 15–30 min at RT. The reaction was stopped with ELISA Stop Solution (Invitrogen, Thermo Fisher Scientific, Waltham, MA, USA), and the acquisition was performed at 450 nm using VICTOR^®^ Nivo™ Plate Reader (PerkinElmer, Milan, Italy) and VICTOR^®^ Nivo™ Control Software (v 4.0.7, PerkinElmer, Milan, Italy). End-point titers were calculated as the last dilution with an optical density (O.D.) ≥ 1.5 blank for IgG and ≥ 3 blank O for IgM and IgA. Specificity was evaluated using a competition assay as the percentage of reduction in O.D. value of serum samples diluted 1:100 untreated or pre-incubated with SARS-CoV-2 S1 RBD recombinant protein (5 µg/mL for IgG and IgA, 30 µg/mL for IgM) for 1 h at 37 °C.

Neutralizing antibody test was performed with a SARS-CoV-2 Neutralizing Antibody ELISA Kit (Invitrogen, Thermo Fisher Scientific, Waltham, MA, USA) as previously described [19] and following the manufacturer’s instructions. For that, serum samples were diluted 1:50 with Assay Buffer 1X, and data were acquired at 450 nm, using 620 nm as the reference wavelength, with a VICTOR^®^ Nivo™ Plate Reader (PerkinElmer, Milan, Italy) using VICTOR^®^ Nivo™ Control Software (v 4.0.7, PerkinElmer, Milan, Italy). The percentage of neutralization was calculated using the following formula: 1 − (absorbance of unknown sample/absorbance of negative control) × 100. Data presented refer to n = 5 for rats treated with oEVs and n = 6 for rats treated with mRNA-loaded oEVs.

### 2.8. IFN-γ Detection with Elispot

ELISPOT assays were performed using Rat IFNγ ELISPOT Set (Abcam, Cambridge, UK) following the procedure previously described [19]. Briefly, ELISPOT plates (Merck Millipore, Darmstadt, Germany) were activated by adding 15 µL of 35% ethanol to each well. The coating with rat IFN-γ ELISPOT Capture Antibody was incubated overnight at + 4 °C. Plates were blocked with RPMI 1640 with 10% FBS for 2 h at RT. Freshly isolated rat splenocytes were plated at the concentration of 3 × 10^5^ cells/well and stimulated with 2 μg/mL S peptide (SARS-CoV-2 S1 RBD recombinant protein, RP-87706, Thermo Fisher Scientific, Waltham, MA, USA). Each condition was repeated for three technical replicates. Plates were incubated for 44 h at 37 °C with 5% CO_2_. Then cells were removed, and the assay was developed following the manufacturer’s instructions. The substrate was incubated for 20 min until spot development; then, the reaction was stopped by washing with sterile water, and plates were let air-dry at RT in the dark. Plate acquisition was performed with the ImmunoSpot plate reader (S6 Macro M2, ImmunoSpot, Cleveland, OH, USA), and spot count was performed automatically by ImmunoSpot Software version 7.0.21.0 (ImmunoSpot, Cleveland, OH, USA). Data presented refer to n = 5 for rats treated with oEVs and n = 6 for rats treated with mRNA-loaded oEVs.

### 2.9. Cytokine ELISA Analysis

Rat IFN-γ, IL-2, IL-4, and IL-10 were quantitatively detected with IFN-γ, IL-2, IL-4 Rat ELISA Kit and Rat IL-10 Uncoated ELISA Kit following manufacturer’s instructions (Thermo Fisher Scientific, Waltham, MA, USA). Briefly, rat splenocytes were stimulated with 1 μg/mL of S peptide for 24 h, and the supernatant was collected and centrifuged to remove debris at 1400× *g* for 10 min before analysis. Plates were coated with capture antibody overnight at +4 °C and blocked with ELISA/ELISPOT Diluent 1X for 1 h at RT (Thermo Fisher Scientific, Waltham, MA, USA). Rat cytokine standards were reconstituted and serially diluted to make the standard curve for a total of 8 points. ELISA/ELISPOT Diluent was used as blank, whereas cell supernatant was used in other wells. Plates were read at 450 nm subtracting 570 nm reference wavelength on VICTOR^®^ Nivo™ Plate Reader (PerkinElmer, Milan, Italy) using VICTOR^®^ Nivo™ Control Software version 4.0.7 (PerkinElmer, Milan, Italy). Standard curves based on standard O.D. values were used to calculate the cytokine amount (pg/mL) for each sample. Data presented refer to n = 5 for rats treated with oEVs and n = 6 for rats treated with mRNA-loaded oEVs.

### 2.10. Cytofluorometric Analysis of Splenocyte-Derived Immune Cells

Rats splenocytes were analyzed for detecting lymphocyte activation markers. Cells were stimulated with 1 μg/mL of S peptide, harvested after 36 h and stained with Viobility Fixable Dye (Miltenyi, Bologna, Italy) and CD3ε-APC-Vio770 (1:50, 130-122-015), CD4-VioBlue^®^ Ex\Em 400\450 (1:50, 130-123-286), CD8-APC (1:50, 130-108-882), CD44-PE (1:10, 130-107-802) (Miltenyi, Bologna, Italy), CD25-FITC (1:50, 561783, BD Bioscience, Eysins, Switzerland), CD138 (0.8 µg/test, MA5-44041, Invitrogen, Thermo Fisher Scientific, Waltham, MA, USA), IL-10-PE (1 µg/test, 555088, BD Bioscience, Eysins, Switzerland), IL-4 (1 µg/test, 555082, BD Bioscience, Eysins, Switzerland), and appropriate isotypes for each fluorescence. For CD138, the secondary antibody Goat anti-Mouse IgG Fc Cross-Adsorbed Secondary Antibody, APC (1:50, 31981, Thermo Fisher Scientific, Waltham, MA, USA) was incubated for 1 h. After staining, cells were washed with PBS, centrifuged at 300× *g* for 10 min, and resuspended in PBS for acquisition. For intracellular cytokine analysis, cells were previously permeabilized using Inside Stain Kit (130-090-477, Miltenyi, Bologna, Italy).

For cell proliferation, splenocytes were stained with CellTrace™ CFSE cell proliferation kit (Invitrogen, Thermo Fisher Scientific, Waltham, MA, USA) before peptide stimulation in vitro, following the manufacturer’s instruction and as previously described [19]. Briefly, 1 × 10^6^ cells were stained with 1 M CellTrace™ stock solution and stimulated with 1 μg/mL of S peptide. Cells were incubated for 5 days and stained with CD3ε APC-Vio^®^ 770 antibody (1:50, 130-122-015, Miltenyi, Bologna, Italy) and the associated isotype.

After washing, cells were analyzed by a CytoFLEX flow cytometer (Beckman Coulter, Milan, Italy) using CytExpert software (v2.3.0.84, Beckman Coulter, Milan, Italy). Data presented refer to n = 5 for rats treated with oEVs and n = 6 for rats treated with mRNA-loaded oEVs.

### 2.11. Intestine Histological Analysis

Morphological analysis of rat intestine was evaluated on jejunum, duodenum, ileum, and colon sections through formalin-fixed paraffin-embedded tissue staining. Paraffin tissue sections (5 μm thick) were routinely stained for microscopic evaluation with H&E (Merck, Darmstadt, Germany) and photographed with an Axioskop microscope (Zeiss, Oberkochen, Germany) equipped with Canon DS126181 camera (Canon, Tokyo, Japan). Data presented refer to n = 5 for rats treated with oEVs and n = 6 for rats treated with mRNA-loaded oEVs.

### 2.12. Statistical Analysis

Data analysis was performed using GraphPad Prism 6.0 Demo (GraphPad, San Diego, CA, USA) and was the most appropriate based on the number of groups: three or more groups of data were performed using ANOVA, whereas two groups were compared using *t*-test. Values were expressed as their mean ± SD. Statistical significance was established at *p* < 0.05 (* *p* < 0.05, ** *p* < 0.01, *** *p* < 0.005, and **** *p* < 0.001), whereas ns was used to define no statistical significance (*p* > 0.05).

## 3. Results

### 3.1. Treatment Characterization

**Figure 1:**
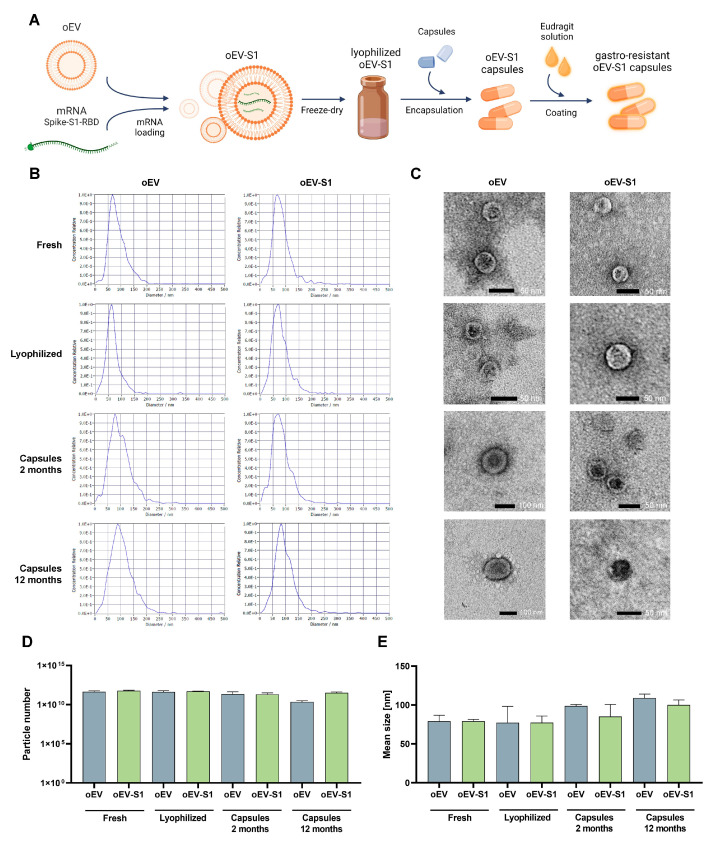
oEV characterization and formulation. (**A**) Schematic representation of treatment production: oEVs were loaded with S1 mRNA, lyophilized, and used to fill oral capsules. Subsequently, capsules were coated to make them gastro-resistant and stored at room temperature (RT). (**B**) Representative size distribution profiles of samples detected via Nanoparticle Tracking Analysis using Zetaview instrument. (**C**) Representative images of transmission electron microscopy (TEM) acquisitions of samples. Scale bar 50 or 100 nm. (**D**) Particle number and (**E**) mean size (nm) measured via Nanoparticle Tracking Analysis using Zetaview instrument. Samples: oEVs unloaded (oEV) or loaded with S1 mRNA (oEV-S1) freshly analyzed (Fresh) or measured after lyophilization (Lyophilized), after lyophilization and storage in oral capsules for 2 months (Capsules 2 months) or 12 months (Capsules 12 months). No statistical differences were detected between samples using one-way ANOVA with Bonferroni’s multiple comparison tests. Data are presented as mean ± SD.

In order to produce oral capsules containing mRNA vaccine, mRNA coding for the S1 antigen of SARS-CoV-2 was loaded into orange-derived EVs (oEVs) as previously documented [19], and the mix was lyophilized before capsules filling and coating, as schematized in Figure 1A. Unloaded oEVs, without mRNA were used as control samples. Particle analysis was performed to verify EV integrity and morphology at different timings of storage: immediately after production; after lyophilization; after 2 months at room temperature (RT) in capsules; and after 12 months at RT in capsules. The light scattering analysis showed a similar size distribution profile in both unloaded oEVs (oEV) and oEVs loaded with S1 mRNA (oEV-S1) after all storage conditions (Figure 1B). Particle morphology was detected using the TEM technique demonstrating an unaltered particle conformation with round morphology and an electron-dense core in all conditions of storage (Figure 1C). Finally, the quantitative analysis of particle number and size did not reveal a significant change after lyophilization, encapsulation, and storage at RT (Figure 1D,E). Importantly, the loading procedure and lyophilization did not significantly alter the number of particles revealing a relative variation of 1.29 ± 0.22 after loading, 1.1 ± 0.09 after loading and lyophilization, and 0.70 ± 0.20 in capsules until twelve months in comparison to unloaded oEVs.

**Figure 2:**
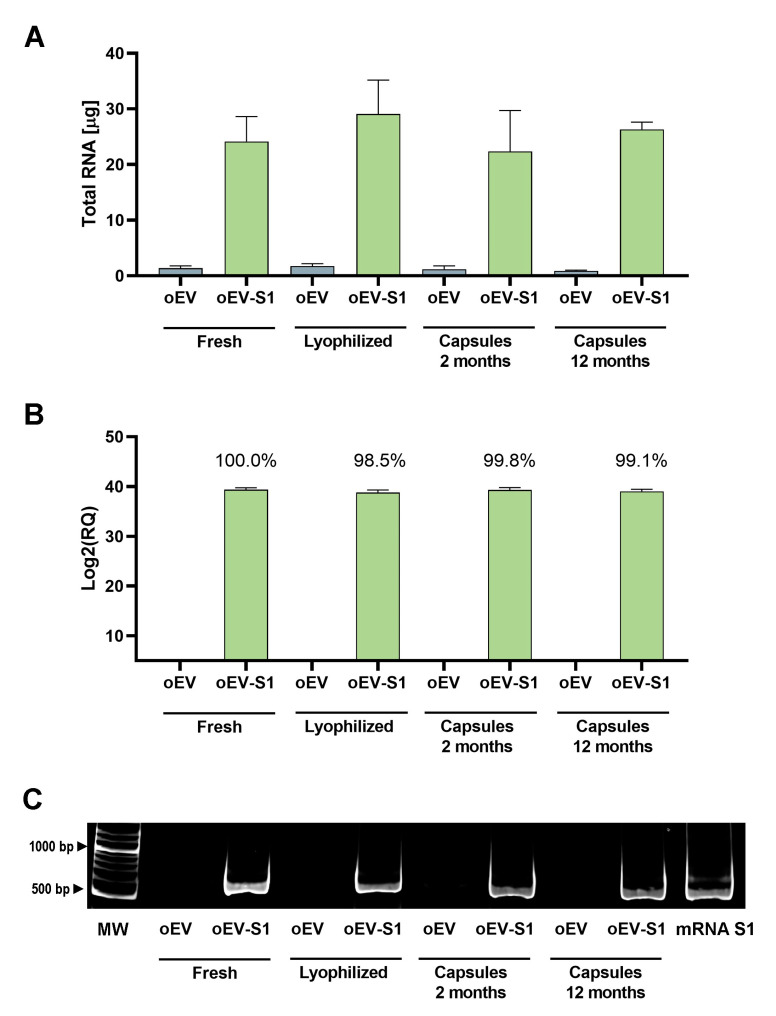
mRNA maintaining over time. (**A**) Total RNA quantification, expressed as total µg. (**B**) S1 mRNA relative quantification was performed using qRT-PCR. The percentage indicates the amount of S1 mRNA that remained at the time point in comparison with the starting amount present in fresh oEV-S1. (**C**) Representative PCR gel showing S1 mRNA in all samples. For all molecular analyses, direct sample comparison was conducted on the same amount (volume) of sample processed for each condition. Samples: DNA molecular weight (MW), oEVs unloaded (oEV) or loaded with S1 mRNA (oEV-S1) freshly analyzed (Fresh) or measured after lyophilization (Lyophilized), after lyophilization and storage in oral capsules for 2 months (Capsules 2 months) or 12 months (Capsules 12 months). No statistical differences were detected between samples using one-way ANOVA with Dunnett’s multiple comparison test. Data are presented as mean ± SD.

The analysis of S1 mRNA loaded into oEVs showed that there was no loss of mRNA comparing oEVs loaded with S1 mRNA (oEV-S1) freshly analyzed or analyzed after lyophilization (lyophilized) or lyophilization and storage in capsules for 2 months (Capsules 2 months) or 12 months (Capsules 12 months). In fact, all conditions demonstrated a similar amount of total RNA (Figure 2A) and of S1 mRNA detected by qRT-PCR (Figure 2B). The S1 mRNA maintenance yield after storage was about 100% in comparison to the starting amount present in freshly prepared samples. The data was also confirmed by PCR assay, showing a similar band intensity in all samples (Figure 2C). As expected, unloaded EVs (oEV) contained less total RNA in comparison with loaded EVs (oEV-S1) and did not express S1 mRNA.

Taken together, these data demonstrated that S1 mRNA loaded into oEVs and oEVs themselves remained stable after lyophilization, encapsulation, and storage at RT until 12 months at least.

### 3.2. Rat Immunization and Antibody Response

**Figure 3:**
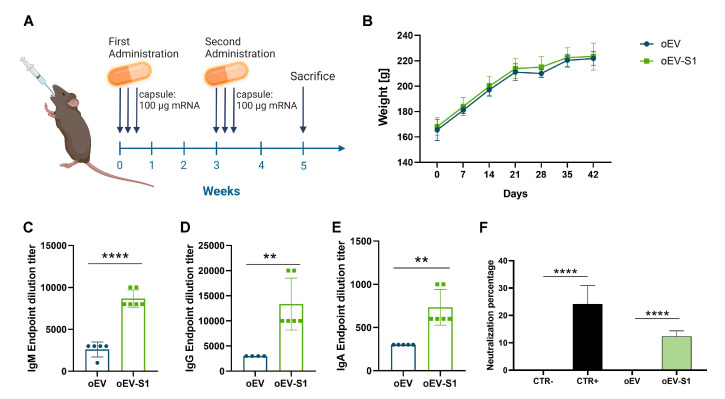
Rat vaccination with oral capsules. (**A**) Schematic representation of immunization treatment. Each rat was treated with a capsule containing 100 µg of S1 mRNA (oEV-S1) or unloaded oEVs (oEV) for three consecutive days, and the treatment was repeated after 3 weeks as a booster dose. Animals were sacrificed after 2 weeks from the last dose and analyzed. (**B**) Animal’s weight measured weekly and expressed in grams (g). (**C**–**E**) Titer of immunoglobulin (Ig) specifically directed against S1 antigen detected in rat serum at the experimental endpoint measured using ELISA assay. Statistical analysis compared the two treatments using *t*-test statistical analysis. (**F**) Percentage of neutralization detected in samples using a competitive ELISA assay for ACE2. Statistical analysis compared negative and positive assay controls (CTR- and CTR+) and samples using ONE-way ANOVA with Tukey’s multiple comparison test. Samples: rats treated with capsules containing unloaded oEVs (oEV) or S1 mRNA-loaded oEVs (oEV-S1). Data are presented as mean ± SD. ** *p* < 0.01, **** *p* < 0.001.

To evaluate the immunization capacity of S1 mRNA loaded into oEVs and administered using oral capsules, female Sprague Dawley rats were treated using a capsule dosing system for gavage following the vaccination protocol summarized in Figure 3A. Rats were vaccinated with the administration of 1 capsule containing oEVs loaded with 100 µg of S1 mRNA (oEV-S1) in three consecutive days, and a booster treatment was repeated after 3 weeks. After 2 additional weeks, animals were sacrificed, and analyses were performed. As control treatments, rats were immunized with capsules containing unloaded oEVs (oEV). Animal weight normally increased after treatments during the entire experimental procedure, suggesting the absence of toxicity of treatments (Figure 3B). This data was also supported by the histological analysis of the intestine showing the absence of morphological alterations (Figure S1). The vaccination with oEV-S1 capsules induced a specific immunoglobulin (Ig) response direct against the S1 antigen, promoting an IgM, IgG, and IgA production (Figure 3C–E) in comparison to the treatment with unloaded oEV capsules. Additionally, serum antibodies produced in rats vaccinated with oEV-S1 capsules were shown to induce neutralization activity, blocking the bond of the S1 protein with the ACE2 receptor (Figure 3F). The specificity of the antibodies for the S1 protein was confirmed by the absence of detectable antibodies in sera before vaccination (Figure S2A–C) and by the ELISA specificity assay (Figure S2D–F).

### 3.3. Splenic Immune Activation

**Figure 4:**
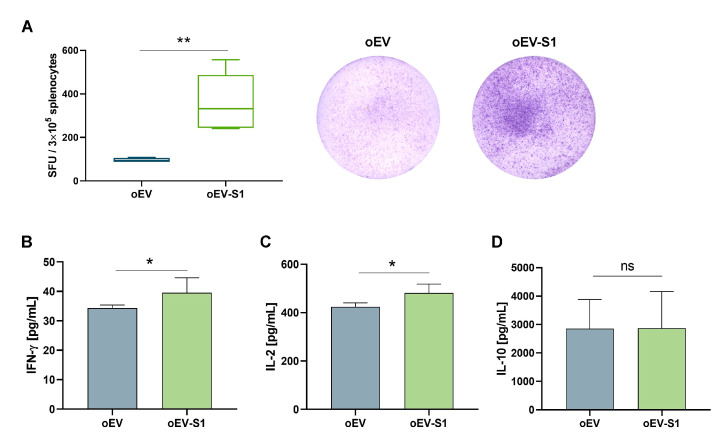
Cytokine secretion from splenocytes isolated from vaccinated rats. (**A**) IFN-γ secretion was measured using ELISPOT assay and expressed as spot-forming units (SFU) per 3 × 10^5^ cells. Representative spot images are shown for each treatment. (**B**–**D**) Cytokine secretion by splenocytes after 24 h of stimulation with SARS-CoV-2 S1 peptide quantified by ELISA assay and expressed as pg/mL for IFN-γ (**B**), IL-2 (**C**), and IL-10 (**D**). Samples: rats treated with capsules containing unloaded oEVs (oEV) or S1 mRNA-loaded oEVs (oEV-S1). Data are presented as mean ± SD and were compared using *t*-test statistical analysis. ns, not statistically significant. * *p* < 0.05, ** *p* < 0.01.

The immune activation induced by vaccination was evaluated on splenocytes isolated from rats stimulated with S1 antigens ex vivo. The treatment with oEV-S1 capsules induced a Th1 (IFN-γ, IL-2) instead of Th2 (IL-10, IL-4) cell-mediated immune response. In fact, a strong IFN-γ secretion was detected by ELISPOT assay (Figure 4A) and confirmed by ELISA assay (Figure 4B) in splenocytes isolated from rats vaccinated (oEV-S1) in comparison to control (oEV). Furthermore, increased secretion of IL-2 (Figure 4C) but not IL-10 (Figure 4D) was measured by splenocytes of vaccinated rats, whereas IL-4 was not detected using ELISA assay.

**Figure 5:**
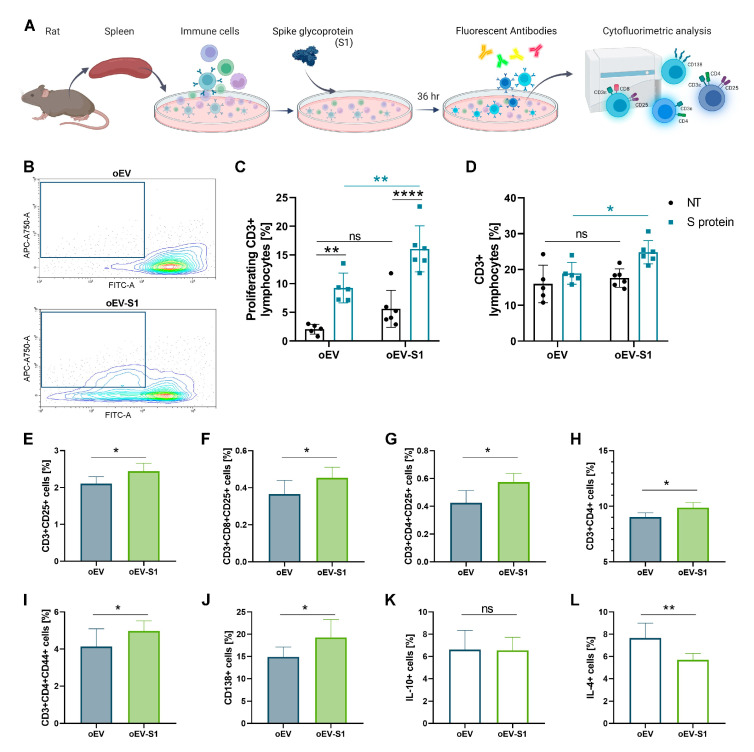
Rat vaccination with oral capsules. (**A**) Schematic representation of the experimental workflow: cells were isolated from rat spleen and stimulated or not with S1 antigen ex vivo before cytofluorimetric analysis. (**B**) Representative plots of splenocytes isolated from rats treated with oEV or oEV-S1 and analyzed for CD3+ proliferation using CFSE assay. The boxes highlight proliferating cells. (**C**) Quantitative analysis of proliferating CD3+ lymphocytes in samples untreated (NT) or treated with S1 antigen (S protein) expressed as percentage of events detected using CFSE assay. Samples were compared using TWO-way ANOVA with Tukey’s multiple comparison test. (**D**) Cytofluorimetric quantification of total CD3+ lymphocytes in samples untreated (NT) or treated with S1 antigen (S protein) expressed as percentage of events. (**E**–**L**) Analysis of splenocyte immune populations expressed as the percentage of events detected for CD3+CD25+ (**E**), CD3+CD8+CD25+ (**F**), CD3+CD4+CD25+ (**G**), CD3+CD4+ (**H**), CD3+CD4+CD44+ (**I**), CD138+ (**J**), and IL-10+ (**K**), IL-4+ (**L**). Samples: rats treated with capsules containing unloaded oEVs (oEV) or S1 mRNA-loaded oEVs (oEV-S1). Data are presented as mean ± SD and were compared using *t*-test statistical analysis. ns, not statistically significant. * *p* < 0.05, ** *p* < 0.01, **** *p* < 0.001.

Splenocytes isolated from rats and stimulated or not with S1 antigen ex vivo were also investigated using cytofluorimetric analysis (Figure 5A). Rats vaccinated with oEV-S1 demonstrated an increased amount of proliferating CD3+ lymphocytes in comparison to oEV when stimulated in vitro with S1 antigen, but not without treatment (Figure 5B,C). As expected, antigen stimulation induced lymphocyte activation in vitro, but lymphocyte response was clearly superior in rats vaccinated and treated with oEV-S1 in comparison to oEV. Consequently, the total number of CD3+ lymphocytes was higher following stimulation with S1 antigen in splenocytes isolated from vaccinated rats compared to control animals (Figure 5D). Immunization with oEV-S1 also induced an adaptive response in comparison to treatment with unloaded oEV with the promotion of CD25+ expression in both total CD3+ lymphocytes and CD3+CD4+ and CD3+CD8+ cells (Figure 5E–G). Moreover, an increased level of CD3+CD4+ cells and their expression of the activation marker CD44+ was detected after vaccination (Figure 5H,I). The immunization with oEV-S1 was able to promote the formation of CD138+ plasma cells (Figure 5J). Finally, cytofluorimetric analysis confirmed the unchanged level of IL-10 and revealed the reduction in IL-4 secretion following vaccination with oEV-S1 (Figure 5K,L).

## 4. Discussion

The present study demonstrated that plant-derived EVs can be efficiently used as carriers of mRNA vaccines and simply formulated in easy-to-use oral capsules. EVs are emerging as promising technology for drug delivery thanks to their native ability to protect and transfer their cargo to target cells. With respect to synthetic nanoparticles, EVs are better internalized by cells and have demonstrated low or absent toxicity [20]. In addition, it has been shown that infected cells may release EVs carrying viral antigens able to initiate an immune response [21,22] and that circulating EVs carrying S protein were detected in infected patients, suggesting a possible involvement in immune response [23]. Human cell-derived EVs were proved to be suitable for the encapsulation of therapeutic agents either directly or by manipulation of the cell of origin [24]. Notably, it has been documented that the antigens associated with EVs were more efficient than soluble antigens in triggering cytotoxic T-cell-mediated response. [25]. However, the use of human cell-derived EVs for vaccine development is limited due to the difficulty and cost of large-scale production.

An alternative to human cell lines for mRNA vaccine delivery is the use of plant EVs. This strategy is particularly attractive because plant EVs are an extractive product that can be produced on a large scale with high yields; they are nontoxic, protect nucleic acids from stress and enzyme degradation, and can be directly manipulated [26,27,28,29]. Moreover, those derived from edible plants also have good gastrointestinal tolerance, stability, and absence of immunogenicity. It has been suggested that plant EVs may be exploited not only for the delivery of microRNAs (miRNAs), small interfering RNAs (siRNAs), and DNA but also for poorly soluble compounds [27]. In this study, we verified for the first time the use of orange-derived EVs as a delivery system of an mRNA-based vaccine formulated in oral capsules.

Here, we demonstrated that oEVs carrying SARS-CoV-2 S1 mRNA preserve the functional integrity of mRNA after lyophilization. In fact, the natural membrane of EVs protects from stress conditions related to lyophilization and confers stability to mRNA until 1 year, allowing encapsulation of the powder in edible gastro-resistant capsules. Accordingly, we recently showed that oEVs protect nucleic acid from RNase, acid gastric pH, and digestive enzymes [19]. Previously, we demonstrated the efficacy of SARS-CoV-2 mRNA carried by oEVs in soluble formulation to trigger a specific humoral and cellular immune response after intramuscular injection and oral and intranasal administration [19]. In this study, EVs were loaded into gastro-resistant capsules, which were dissolved in the first intestinal tract, where most of the immune system is located, preventing gastro-dispersion [30]. The oral administration of capsule formulation induced rat immunization triggering a cellular and humoral immune response with the production of neutralizing antibodies and specific IgM, IgG, and IgA. The IgA represent the first mucosal barrier in the adaptive immune response, and their stimulation is at the basis of mucosal vaccination [31,32]. However, mucosal vaccination not only stimulates IgA production but also high level of serum antigen-specific IgM and IgG, thus providing systemic protection [33]. Interestingly, among the antibody response to SARS-CoV-2 infection, IgA antibodies contributed more to virus neutralization than IgG in patients [34]. Interestingly, mucosal vaccination obtained by direct inhalation of mammalian EVs loaded by electroporation with S protein mRNA showed to be significantly superior to synthetic liposomes used as mRNA carriers [35]. Moreover, vaccines delivered orally can directly reach the site where the majority of the immune system resides, allowing an efficient immune response. The rat immunization did not show toxicity, as demonstrated by animal behavior and histological analysis. We showed that the rat vaccination also induced immune cell activation with a Th-1 cytokine secretion profile, with an increased release of IFN-γ and IL-2 but not of IL-10 or IL-4. Moreover, immunization with oEV loaded with S1 mRNA was able to stimulate splenic CD3+ lymphocyte proliferation and the expression of activation marker CD25+ on both total CD3+ lymphocytes and CD4+ and CD8+ lymphocytes [36]. Besides the promotion of CD138+ plasma cells [37], an increase in CD3+CD4+ T helper and CD4+CD44+ activated memory T cells was also detectable following vaccination, supporting lymphocyte activation [38]. These data are consistent with previous work demonstrating that the oral and intranasal immunization with food-grade recombinant Lactococcus lactis expressing SARS-CoV-2 spike protein promoted an immune response in mice with the increase in CD138+ plasma cells [39]. Recently, Lei et al. showed how intranasal immunization with RBD antigen and polyethyleneimine increased the percentage of memory CD4+ T cells (CD4+ CD44+) in the lungs when compared with immunization with PBS or RBD alone [40]. Moreover, the mucosal vaccination for SARS-CoV-2 was demonstrated to elicit an increased CD4+ T cell response in mice [41].

In conclusion, EVs extracted from edible plants may represent a platform for oral vaccine delivery for their optimal mucosal absorption and easy formulation with oral edible capsules. The SARS-CoV-2 S1 mRNA-based EV vaccine can be considered a prototype for the development of vaccines against several other infectious diseases, showing the potentiality of plant EVs. In fact, the formulation used in this study was not fully optimized for an efficient in vivo translation and immunization, reducing variables that affect vaccination and allowing for the evaluation of the oEV raw effect. The formulation improvements by using mRNA sequence optimized for an efficient in vivo translation and by adding appropriate adjuvants to stimulate immune response would additionally increase the immunization effect and reduce mRNA dose. Here, for the first time, it has been demonstrated that plant-derived EVs can be an efficient and practical carrier for an mRNA-based vaccine using a formulation easily transferable to the clinics. The oral vaccination directly targets the largest compartment of the immune system in the body and the intestine, allowing for an efficient immunization. Furthermore, oral administration represents a needle-free treatment that could improve large-scale vaccination campaigns by increasing ease and speed and reducing costs and pain associated with vaccination. To better define the translational potential of oEVs, the direct comparison of oEVs with liponanoparticles currently used for mRNA vaccine delivery and a deep investigation of oEV toxicity profile would be useful to define their potential as delivery systems.

## Data Availability

Data are contained within the article.

## Supplementary Materials

The following supporting information can be downloaded at: https://www.mdpi.com/article/10.3390/cells12141826/s1, Figure S1: Histological analysis of rat intestine; Figure S2: ELISA specificity for immunoglobulin response measurement in rat serum. Table S1: Primer sequences used for qRT-PCR and PCR experiments.
