# Supplementary material for: Oral Delivery of mRNA Vaccine by Plant-Derived Extracellular Vesicle Carriers
Source: Cells. 2023 Jul 11;12(14):1826. doi: 10.3390/cells12141826 (PMC10378442; doi:10.3390/cells12141826)
Supplement: Supplementary file 1 [file cells-12-01826-s001.zip › cells-2476639-supplementary.pdf]

Supplementary Information

Oral delivery of mRNA vaccine by plant-derived extracellular vesicle carriers

Margherita A. C. Pomatto [1,2,*], Chiara Gai [1,2], Federica Negro [1], Lucia Massari [1], Maria Chiara Deregibus [2], Francesco Giuseppe De Rosa [2] and Giovanni Camussi [1,2,*]

[1] EvoBiotech s.r.l., 10122 Turin, Italy; cgai@evobiotech.it (C.G.); fnegro@evobiotech.it (F.N.); lmassari@evobiotech.it (L.M.)

[2] Department of Medical Science, University of Turin, A.O.U. Città della Salute e della Scienza di Torino, 10126 Turin, Italy; mariachiara.deregibus@unito.it (M.C.D.); francescogiuseppe.derosa@unito.it (F.G.D.R.)

* Correspondence: mpomatto@evobiotech.it (M.A.C.P.); giovanni.camussi@unito.it (G.C.)

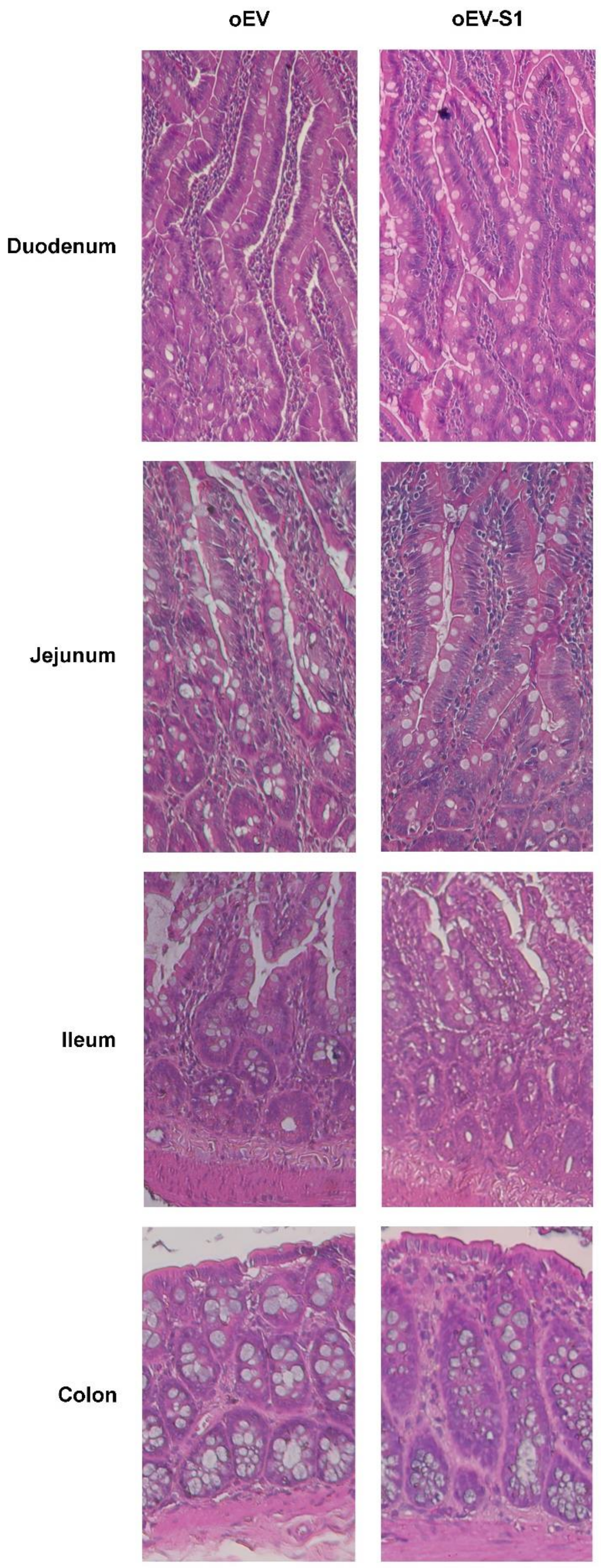

Figure S1. Histological analysis of rat intestine. H&E histological representative images of main sections of rat intestine (duodenum, jejunum, ileum, and colon) at the experimental endpoint after the vaccination with capsules containing unloaded oEVs (oEV) or S1 mRNA loaded oEVs (oEV-S1). 20X magnification.

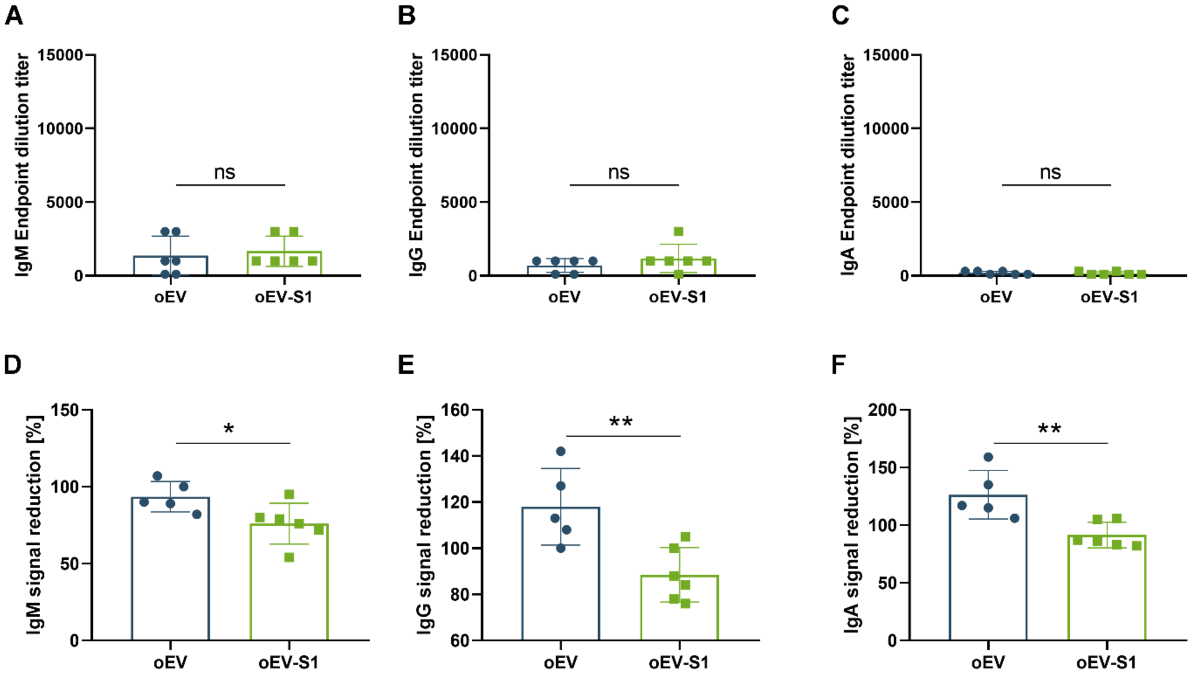

Figure S2. ELISA specificity for immunoglobulin response measurement in rat serum. (**a-c**) Antibody titer was detected in serum at day 0 before vaccination for IgM (**a**), IgG (**b**), and IgA (**c**). (**d-f**) Competition assay to determine ELISA specificity. Serum samples were pre-incubated with an excess of S1 protein to verify the reduction of only specific signals for IgM (**d**), IgG (**e**), and IgA (**f**). Samples: rats treated with capsules containing unloaded oEVs (oEV) or S1 mRNA loaded oEVs (oEV-S1). Data are presented as mean ± SD and compared using t-test statistical analysis. ns, not statistically significant. * $p<0.05$, ** $p < 0.01$, **** $p < 0.001$.

Table S1. Primer sequences used for qRT-PCR and PCR experiments.

Name	Forward Primer (F)	Reverse Primer (R)	Application
cel-mir-39	CACCGGGTGTAAATCAGCTTG	-	qRT-PCR
S1	GCCGGTAGCACACCTTGTAA	ACACCTGTGCCTGTTAAACCA	qRT-PCR
S1	TGTGCCCTTTTGGTGAAGTTTT	TTAGGTCCACAAACAGTTGCT	PCR
